# Thalidomide Combined With Azathioprine as Induction and Maintenance Therapy for Azathioprine-Refractory Crohn's Disease Patients

**DOI:** 10.3389/fmed.2020.557986

**Published:** 2020-11-06

**Authors:** Tong Li, Yun Qiu, Xiaozhi Li, Xiaojun Zhuang, Shanshan Huang, Manying Li, Rui Feng, Baili Chen, Yao He, Zhirong Zeng, Minhu Chen, Shenghong Zhang

**Affiliations:** ^1^Department of Gastroenterology and Hepatology, The First Affiliated Hospital of Sun Yat-sen University, Guangzhou, China; ^2^Department of Medical Ultrasonics, The First Affiliated Hospital of Sun Yat-sen University, Guangzhou, China

**Keywords:** Crohn disease, azathioprine, thalidomide, loss of response, remission

## Abstract

The combination therapy of thalidomide and azathioprine (AZA) offers an alternative in clinical practice for Crohn's disease (CD) patients experiencing a loss of response to AZA monotherapy. However, little is known about the efficacy and safety of this combination therapy for patients with CD. This was a retrospective study of 122 consecutive CD patients who lost response to AZA therapy and had switched to a combination therapy of thalidomide and AZA. The primary outcomes were clinical response and clinical remission rates at week 24. Patients who had an initial response to combination therapy were continued on the treatment for remission maintenance. The secondary outcomes were the proportion of clinical relapse throughout maintenance. The Kaplan–Meier method was used to calculate cumulative rates, and Cox regression analysis was used for multivariate analysis. During induction, 80.3% (98/122) patients achieved clinical response within a median duration of 6.5 weeks, (interquartile range, 4.3–8.1 weeks). The rate of clinical remission at 24 weeks was 70.5%. During follow-up, 22.4% (22/98) of the patients that were maintained on combination therapy experienced clinical relapse. The proportions of patients in remission status at 12, 24, and 36 months were 85.1, 78.3, and 70.1%, respectively. Multivariate analysis revealed C-reactive protein >10 mg/L at disease relapse on AZA monotherapy [adjusted hazard ratio (HR), 4.72; 95% CI, 1.19–18.75, *P* = 0.027] and 6-thioguanine nucleotides level ≥235 pmol/8 × 10^8^ erythrocytes at AZA monotherapy (adjusted HR, 5.32; 95% CI, 1.40–20.14, *P* = 0.014) were associated with disease relapse on combination therapy. The endoscopic remission rate was 63.6%. Mucosal healing was achieved in 23.6% of the patients. Both Crohn's Disease Endoscopic Index of Severity (13.4 ± 4.92 *vs*. 6.12 ± 5.24, *P* < 0.001) and Rutgeerts scores (3.23 ± 0.73 *vs*. 1.77 ± 1.59, *P* = 0.003) were significantly decreased with the use of combination therapy. Adverse events occurred in 62 (50.8%) patients, but only 13 (10.7%) necessitated therapy discontinuation. Thalidomide combined with AZA was effective in inducing clinical remission and sustaining long-term steroid-free remission in CD patients who lost response to AZA monotherapy.

## Introduction

Crohn's disease (CD), an inflammatory bowel disease (IBD) with unknown etiology, may involve any part of the gastrointestinal (GI) tract. The clinical course of CD is characterized by its propensity to relapse (1, 2). Thiopurines, comprising azathioprine (AZA) and mercaptopurine, are the established first-line immunosuppressive therapies with confirmed efficacy for the maintenance of CD remission (3). However, despite the widespread use of thiopurines, 20–40% of CD patients under AZA monotherapy experience a loss of response, requiring optimization or switch to another medication (4, 5). Riello et al. (6) investigated 103 pediatric CD patients on AZA monotherapy and found that the steroid-free remission rates at 6, 12, 18, and 24 months were 60.2, 39.8, 33.3, and 31.2%, respectively. The management of CD patients that are refractory to thiopurines still represents a major concern to GI clinicians.

Current treatment guidelines usually recommend initiating anti-tumor necrosis factor (TNF) agents or methotrexate (MTX) treatment for patients who lost response to AZA therapy (7, 8). However, a total of 20–30% of patients with refractory CD do not respond to anti-TNF-α treatment (9, 10). Similarly, MTX is no more effective than placebo in inducing remission in CD (11); therefore, concomitant steroids are needed for remission induction. A combination of AZA and thalidomide offered an alternative in clinical practice.

Thalidomide is an oral agent that has been reintroduced to treat IBD. It has many immunomodulatory actions, including prevention of angiogenesis, inhibition of TNF-α and interleukin 12, and activation of nuclear factor kappa B and pro-inflammatory cell adhesion (12). Thalidomide has been proven to be effective in the treatment of refractory active CD in both children and adults (12–15) and could be used if biologic agents are unavailable. However, no studies to date have assessed the efficacy and safety of thalidomide combined with AZA in refractory CD.

This retrospective and observational study aimed to assess the efficacy and safety of the combination of thalidomide and AZA in inducing and maintaining clinical and endoscopic remission in AZA-refractory CD patients.

## Materials and Methods

### Study Population

Enrolled patients were screened from among those with established CD diagnosis who were followed up at the Gastroenterology Department of the First Affiliated Hospital, Sun Yat-sen University (China), a tertiary IBD referral center, from May 2004 to December 2019. The diagnosis of CD was based on a combination of clinical, radiologic, endoscopic, and histologic findings. Inclusion criteria were patients (*a*) aged between 16 and 75 years, (*b*) with at least 6 months of steroid-free remission on AZA monotherapy, (*c*) who experienced disease relapse on AZA monotherapy, (*d*) with a mild or moderate disease activity at disease relapse [Crohn's Disease Activity Index (CDAI) between 150 and 450], and (*e*) who switched to a combination therapy of AZA and thalidomide after disease relapse. Exclusion criteria were (*a*) previous thalidomide exposure, (*b*) missing data in the medical records, (*c*) experienced endoscopic relapse but without clinical symptom on AZA monotherapy, and (*d*) follow-up duration <24 weeks.

### Study Design

This is a retrospective single-center study. Patients included were treated with a combination of thalidomide and AZA after losing response to AZA monotherapy. All the patients at our center were fully informed of the risk of teratogenicity and other possible adverse events (AEs) and signed written informed consent before the initiation of thalidomide. Contraception was mandatory for both male and female patients of child-bearing potential throughout the duration of the therapy and up to 6 months after thalidomide had been discontinued. Thalidomide was initiated at a dose of 25 mg/d and increased gradually at a dose of 25–50 mg every 2 weeks until achieving 50–100 mg/d, with close observation of the patients' clinical response and tolerability. Patients with perianal lesion were given antibiotics (levofloxacin and metronidazole) and abscess drainage. Details about all possible AEs of thalidomide, in particular paresthesia or numbness, were enquired from the patients at each visit. Thalidomide was reduced to a lower dose (25–50 mg/d) in patients with neuropathy until this resolved; otherwise, thalidomide may be discontinued at the discretion of the doctor. The diagnosis of neuropathy was largely made based on the typical symptoms of the patients combined with the history of the medication. Electromyography was not regularly performed except for patients with atypical symptoms or relatively severe symptoms.

**Figure 1:**
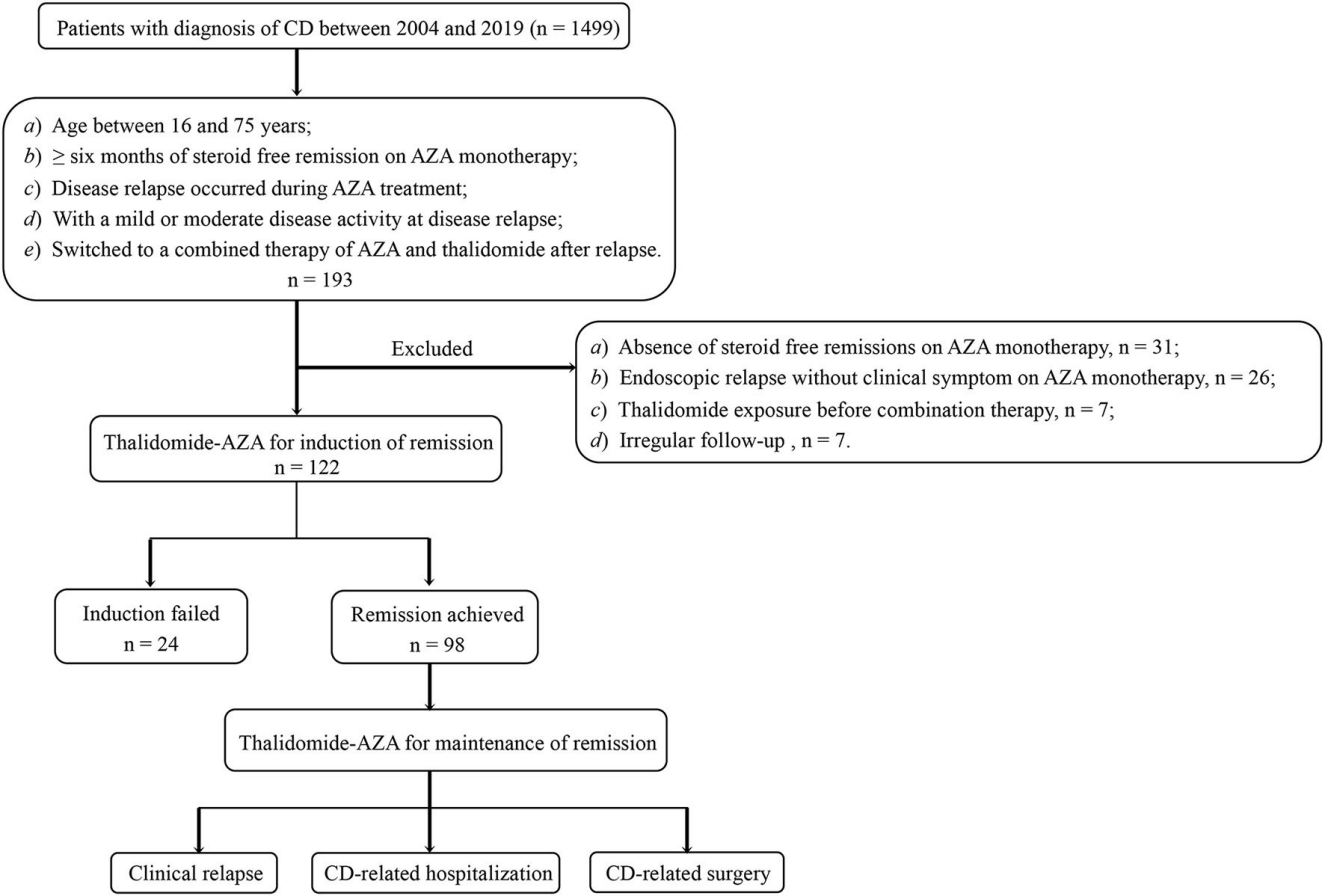
This is the flow of patients in the study. CD, Crohn's disease; AZA, azathioprine.

Patients who had an initial response to the combination therapy at 24 weeks were continued on the treatment for remission maintenance. For patients maintained on the combination therapy, both clinical and endoscopic evaluations were performed. Figure 1 shows the patients' flow in the study. This study was approved by the Ethics Committee of the First Affiliated Hospital of Sun Yat-sen University.

### Definitions and Outcome Measures

The primary efficacy outcomes were the clinical response and remission rates at week 24. The secondary outcomes were the proportion of clinical relapse and CD-related hospitalizations and intestinal surgery throughout the follow-up period of patients maintained on combination therapy.

Loss of response, also referred to as secondary AZA failure, was defined according to a previously validated definition (16, 17) as disease exacerbation after a good initial response (≥6 months steroid independent) to AZA; change in CDAI from remission to active disease, disease relapse between visits, and need for rescue therapy; and thiopurine discontinuation, hospitalization, or surgical intervention.

Clinical remission was defined as a CDAI score <150 with a decrease of >70 points. Clinical response was defined as a CDAI > 150 but with a decrease of >70 points (7). Clinical relapse was defined as a CDAI > 150 with an increase of >70 points (18). A new opening perianal or enterocutaneous fistula, an intra-abdominal abscess, a perianal abscess, a new intestinal obstruction due to CD, confirmed by medical imaging and requiring hospitalization, or endoscopic evidence of active inflammation was also considered to indicate disease relapse (19). CD-related hospitalizations were defined as those resulting from complications or CD-related treatment or adverse reactions (20). CD-related intestinal surgery was defined as any intestinal resection for active CD including the first and subsequent surgeries (21).

Endoscopic remission was defined as Crohn's Disease Endoscopic Index of Severity (CDEIS) score <7 (22) or Rutgeerts score ≤i1 from a baseline Rutgeerts score ≥i2 (23). Mucosal healing (MH) was defined as the absence of ulceration at ileocolonoscopy.

Thalidomide-induced neuropathy was defined when typical subjective and/or objective clinical features were present and associated with electrophysiologic signs and after exclusion of another possible cause of neuropathy. These typical clinical features consisted of pins and needles in the feet and/or tactile distal hypoesthesia, symmetrical, and predominating in the lower limbs (24).

### Adverse Events

Any new symptom or sign, any significant laboratory abnormality, or worsening of a preexisting condition or abnormality that occurred after initiation of combination therapy was considered an AE.

### Data Collection

A predetermined structured datasheet was used to extract data on the following from the medical charts: age at diagnosis, sex, smoking habits, Montreal classification (disease location, and disease behavior), perianal lesions, GI surgery history, 6-thioguanine nucleotides (6-TGN) at AZA monotherapy, dosage of AZA and thalidomide during the combination therapy, and probable AEs. The 6-TGN concentrations were usually measured 8–12 weeks after the initiation of AZA since the steady state may be anticipated following 2 months of use (25). AEs were summarized as numbers and percentages. The hematologic, immunologic, and chemistry laboratory values were also retrieved.

### Statistical Analysis

Statistical analysis was performed using IBM SPSS Statistics for Windows, version 24.0 (IBM Corp., Armonk, N.Y., USA). Quantitative data were described using medians with interquartile range (IQR) while qualitative data were expressed as counts and percentages. Fisher's exact test and chi-squared tests were used to compare non-parametric categorical data between groups, and analysis of variance was used for continuous parameters. The Kaplan–Meier method was used for survival data to calculate the cumulative probabilities of being free of events (bowel resection, hospitalization, or disease flare). Cox regression analysis was used to identify risk factors for disease flare and endoscopic remission. Univariate analysis was performed using the log-rank test to identify independent predictive factors, and factors, with *P* < 0.2 tested in a multivariate analysis using Cox regression. All statistical tests were two-sided. *P* < 0.05 was considered statistically significant.

## Results

### Patients' Characteristics at Baseline

We reviewed the medical charts of 1499 patients with CD referred to our department between 2004 and 2019. Of a total of 193 cases who met the inclusion criteria, 71 cases were further excluded (31 for absence of steroid-free remissions on AZA monotherapy, 26 for endoscopic relapse but without clinical symptom on AZA monotherapy, 7 for thalidomide exposure before combination therapy, and 7 for irregular follow-up). The remaining 122 patients were enrolled (male, 87; female, 35; median age, 30.0 years), 46.7% of the patients were with stricturing disease, and 28.7% had perianal lesion. Fifty-two patients underwent GI surgery, while 10 patients underwent appendectomy. The combination therapy was started at a median time of 20.3 months (IQR 11.7–59.5 months) post-operation. Of 28 patients with upper GI tract involvement, 12 patients had GI surgery history (9 patients had small bowel resection, 1 had ileocolic resection, 1 had gastrojejunostomy, whereas, endoscopic dilation was performed on only 1 patient). Table 1 shows the baseline characteristics of this cohort. The median duration of AZA monotherapy was 18.2 months (IQR, 11.2–35.2 months). 6-TGN levels were measured at a median time of 3.1 months (IQR 0.5–7.8 months) before losing response to AZA, with a median level of 282.2 pmol/8 × 10^8^ erythrocytes, which is within the target therapeutic window [>235 pmol/8 × 10^8^ erythrocytes (26)]. Among all the patients included in our study, none were pregnant, nursing, or undergoing pregnancy preparation.

**Table 1:** Baseline demographic and clinical characters.

**Variable**	***n* = 122**
Gender (male)	87 (71.3)
BMI	19.21 ± 3.24
Smoker	19 (15.6)
Age at referral (years)	30.0 (23.0–39.0)
<40 years	99 (81.1)
**Disease location**^†^	
Ileal (L1)	19 (15.6)
Colonic (L2)	12 (9.8)
Ileocolonic (L3)	89 (73.0)
Upper digestive tract (L4)	28 (23.0)
**Disease behavior**^†^	
Non-penetrating, non-stricturing (B1)	36 (29.5)
Stricturing (B2)	57 (46.7)
Penetrating (B3)	29 (23.8)
Perianal lesion	35 (28.7)
Fistula	23 (18.9)
Perianal abscess	12 (9.8)
Anal Fissure	2 (1.6)
History of GI surgery	52 (42.6)
Partial enterectomy	26 (21.3)
Ileocolic resection ± ileocolic anastomosis	13 (10.7)
Hemicolectomy	7 (5.7)
Ileocecoectomy	5 (4.1)
Gastrojejunostomy	1 (0.8)
History of appendectomy	10 (8.2)
History of biological therapy	33 (27.0)
Disease duration at LOR to AZA (months)	58.5 (28.3–99.8)
Time of remission on AZA monotherapy (months)	18.2 (11.2–35.2)
CDAI at LOR to AZA	204.1 ± 57.4
CRP at LOR to AZA (mg/L)	15.0 (7.6–25.2)
ESR at LOR to AZA (mm/h)	47.5 (25.8–76.7)
6-TGN level on AZA monotherapy (pmol/8 × 10^8^ erythrocyte)	282.2 (185.7–358.2)
6-TGN level on combination therapy (pmol/8 × 10^8^ erythrocyte)	194.4 (132.4–273.9)

† *Phenotypes were categorized using the Montreal classification*.

*The values are described as number (%), mean ± SD or median (IQR)*.

*BMI, body mass index; LOR, loss of response; CDAI, Crohn's disease activity index; CRP, C-reactive protein; ESR, erythrocyte sedimentation rate; GI, gastrointestinal; AZA, azathioprine; TGN, thioguanine nucleotides; SD, standard deviation; IQR, interquartile range*.

### Induction With Thalidomide and AZA

Median follow-up of combination therapy was 33.4 months (IQR, 12.6–63.7 months). AZA was prescribed at a dosage of 2.0–2.5 mg/kg/d and thalidomide 25 mg/d at the start of the combination therapy. Thalidomide dosage was increased to 75 or 100 mg/d based on the level of the patients' tolerance and clinical symptoms. No statistical differences were observed in AZA dosage between combination therapy and previous monotherapy (*P* = 0.23).

**Figure 2:**
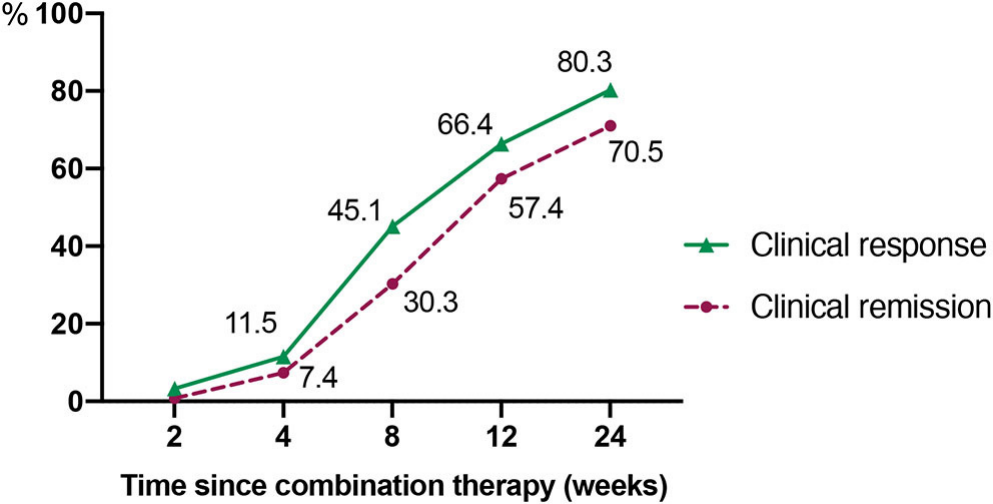
This figure shows the clinical remission and response rates over time.

As demonstrated in Figure 2, at week 24, 80.3% (98/122) of the patients achieved clinical response within a median time of 6.5 weeks (IQR, 4.3–8.1 weeks). The proportions of patients who had a clinical response at week 8 and 12 were 45.1 and 66.4%, respectively. Accordingly, the clinical remission rates at week 8, 12, and 24 were 30.3, 57.4, and 70.5%, respectively. The median time of achieving clinical remission was 9.0 weeks (IQR of 6.0–13.0 weeks). Moreover, there was a significant decrease in CDAI, erythrocyte sedimentation rate (ESR), and C-reactive protein (CRP) at week 8 when compared to the baseline (*P* < 0.001, *P* = 0.039, and *P* = 0.032, respectively) (Table 2).

**Table 2:** Outcomes of thalidomide and AZA combination therapy over time.

	**Week 0**	**Week 4**	**Week 8**	**Week 12**	**Week 24**
Clinical response^1^, *n* (%)	–	14 (11.5%)	55 (45.1%)	81 (66.4%)	98 (80.3%)
Clinical remission^2^, *n* (%)	–	9 (7.4%)	37 (30.3%)	70 (57.4%)	86 (70.5%)
CDAI, mean (SD)	204.1 ± 57.4	162.3 (50.4)^*^	132.1 (47.5)^**^	109.5 (39.1)^**^	84.2 (40.8)^**^
CRP^3^, median (IQR), mg/L	15.0 (7.6–25.2)	11.3 (3.9–18.1)	8.3 (2.9–22.7)^*^	5.7 (1.7–9.2)^**^	2.9 (0.95–6.7)^**^
ESR^4^, median (IQR), mm/h	47.5 (25.8–76.7)	40.0 (23.3–66.5)	37.0 (23.0–58.0)^*^	24.0 (14.0–38.0)^**^	21.0 (13.3–42.0)^**^
WBC^5^, mean (SD), × 10^9^/L	6.6 (2.4)	6.9 (2.2)	6.5 (1.6)	6.1 (2.1)	6.1 (2.6)
Hemoglobin^6^, mean (SD), g/L	124.9 (26.71)	114 (17.9)	117.5 (22.2)	120.5 (22.6)	131.8 (18.1)
PLT^7^, mean (SD), × 10^9^/L	318.8 (101.1)	342.7 (111.8)	339.4 (101.8)	345.7 (132.4)	291.1 (85.0)
BMI, mean (SD), Kg/m^2^	19.21 ± 3.24	19.41 (3.42)	19.91 (2.20)	19.85 (3.34)	20.74 (3.19)^**^

1 *CDAI score decline ≥ 70 from baseline*.

2 *CDAI score < 150 and CDAI score decline ≥ 70 from baseline*.

3 *CRP reference range: 0–3 mg/L*.

4 *ESR reference range: 0–15 mm/h*.

5 *WBC reference range: 4–10 × 10^9^/L*.

6 *Hemoglobin reference range: 130–175 × 10^9^/L*.

7 *PLT reference range: 100–300 × 10^9^/L*.

* *P < 0.05 (compared with baseline)*.

** *P < 0.01 (compared with baseline)*.

*BMI, body mass index; CDAI, Crohn's disease activity index; CRP, C-reactive protein; ESR, erythrocyte sedimentation rate; WBC, white blood cell; PLT, blood platelet; IQR, interquartile range; SD, standard deviation*.

### Maintenance of Remission

#### Clinical Relapse

**Figure 3:**
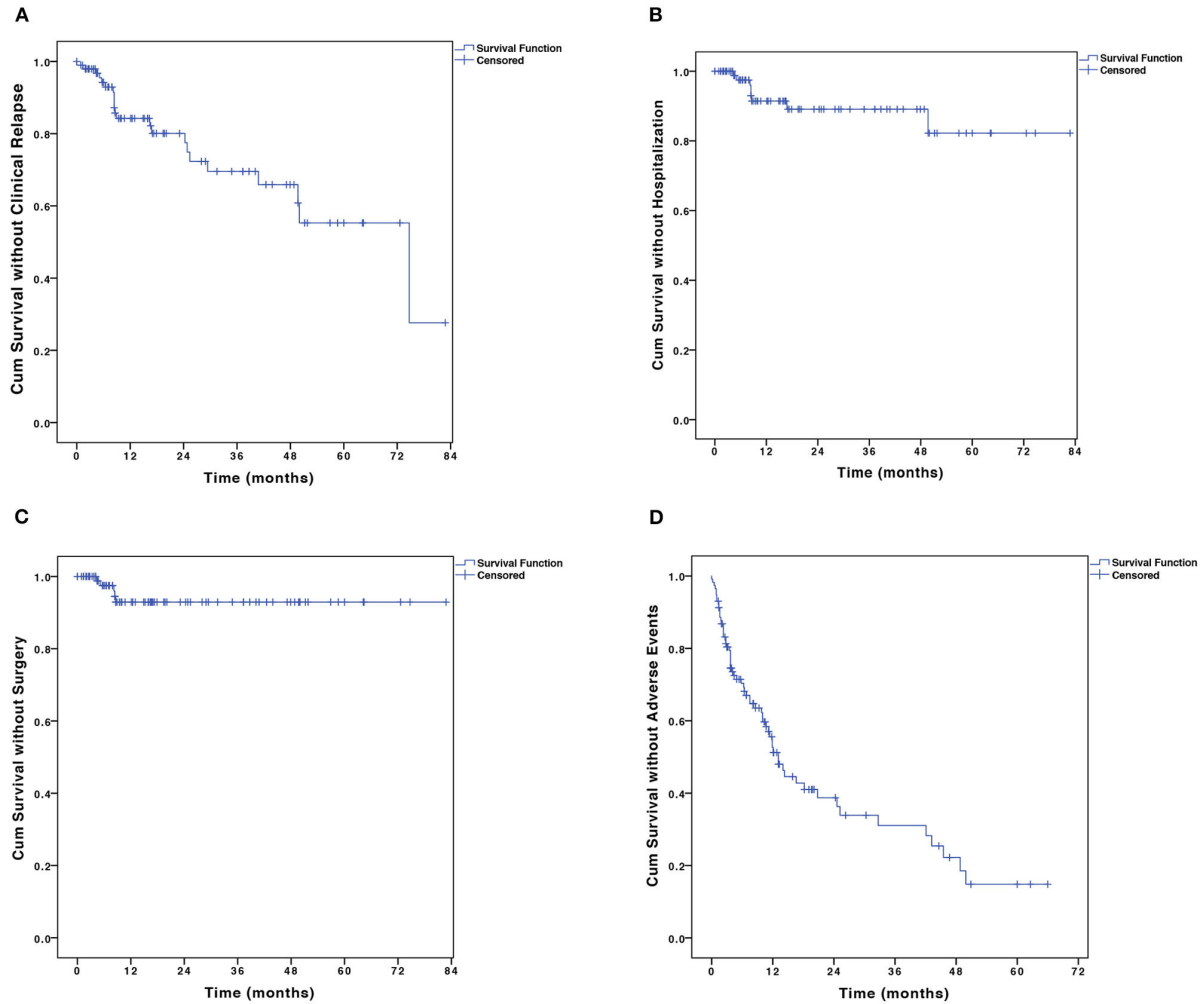
The Kaplan–Meier curve shows the probability of **(A)** disease relapse, **(B)** CD-related hospitalization, **(C)** CD-related bowel surgery, and **(D)** adverse events. CD, Crohn's disease.

Among the 98 patients who achieved initial response and were maintained on combination therapy, 22 (22.4%) patients experienced clinical relapse within a median follow-up time of 38.5 months (IQR, 13.3–66.7 months). The cumulative proportion of patients in remission at 12, 24, 36, 48, and 60 months were 85.1, 78.3, 70.1, 66.6, and 55.8%, respectively (Figure 3A). The median duration of relapse-free survival was 74.7 months [95% confidence interval (CI), 39.6–109.9 months]. The 6-TGN concentrations during combination therapy were measured in 56/98 patients during the maintenance period within a median time of 8.1 months (IQR, 4.7–17.1 months). The median TGN level was 194.4 pmol/8 × 10^8^ erythrocyte (IQR, 132.4–273.9 pmol/8 × 10^8^ erythrocyte).

**Figure 4:**
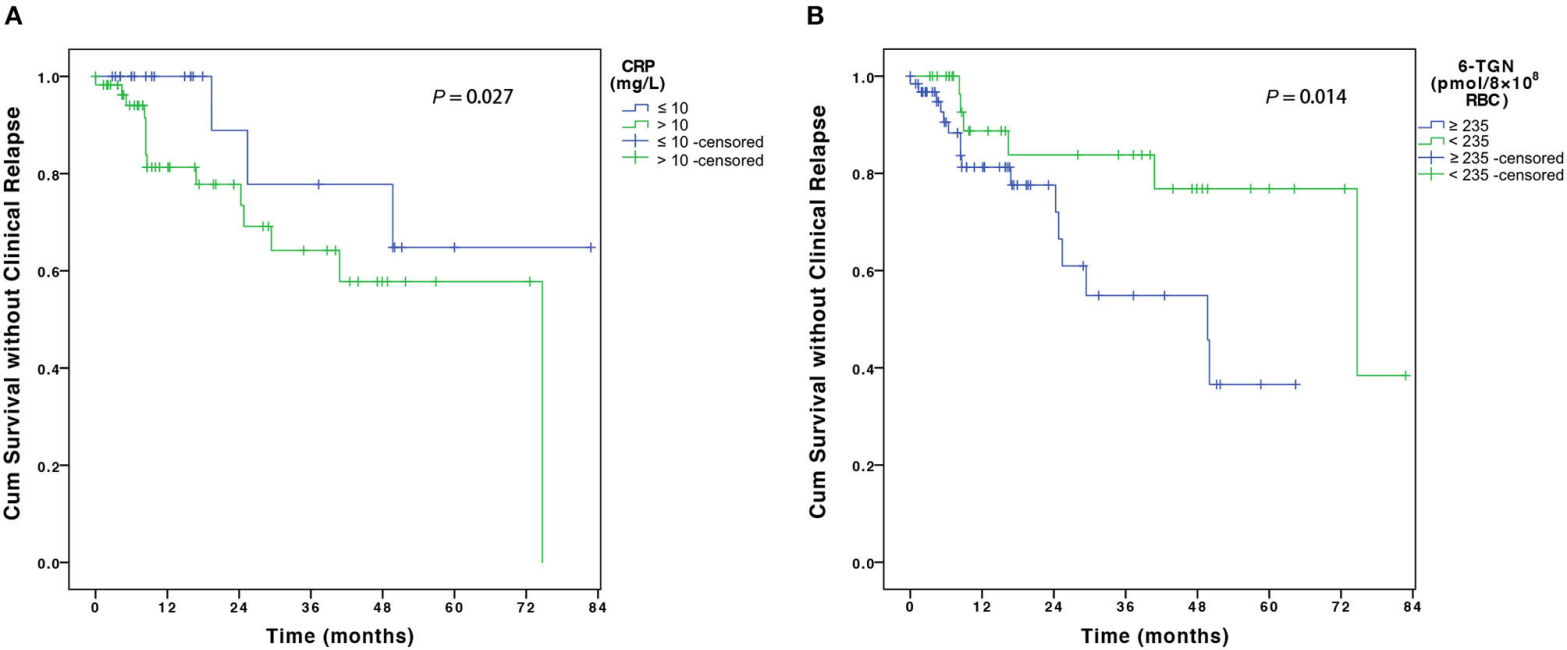
The figure shows the Kaplan–Meier analysis of disease flare according to **(A)** CRP level at disease relapse on AZA monotherapy and **(B)** 6-TGN concentration at AZA monotherapy. CRP, C-reactive protein; AZA, azathioprine; 6-TGN, 6-thioguanine nucleotides.

For risk factors of clinical relapse, all baseline factors that were evaluated in the univariate analysis, including disease behavior, perianal disease, history of GI surgery, ESR, CRP, white blood cell count, and 6-TGN levels showed trends toward influence on disease flare and were tested in multivariate analysis using Cox regression analysis (Table 3). Multivariate analysis revealed that CRP > 10 mg/L at disease relapse on AZA monotherapy [adjusted hazard ratio (HR), 4.72; 95% CI, 1.19–18.75, *P* = 0.027] and 6-TGN level ≥235 pmol/8 × 10^8^ erythrocytes at AZA monotherapy (adjusted HR, 5.32; 95% CI, 1.40–20.14, *P* = 0.014) were associated with disease relapse on combination therapy (Table 3 and Figures 4A,B).

**Table 3:** Predictors of clinical relapse by univariate analysis (log-rank test) and multivariate analysis (Cox model).

**Factors**	**Univariate**		**Multivariate** **Method: Enter**		**Multivariate** **Method: Forward Stepwise**	
	***P***	**HR (95% CI)**	***P***	**HR (95% CI)**	***P***	**HR (95% CI)**
Gender, female	0.354	0.60 (0.20–1.79)				
Age, ≥40 years	0.871	0.90 (0.27–3.07)				
Smoker	0.211	0.04 (0.01–6.68)				
Disease duration	0.565	1.03 (0.94–1.13)				
Disease location^†^	0.705	0.97 (0.8–1.16)				
Ileal (L1)		Reference				
Colonic (L2)	0.749	0.72 (0.10–5.40)				
Ileocolonic (L3)	0.380	0.63 (0.22–1.78)				
Upper digestive tract (L4)	0.994	0.97 (0.39–2.57)				
Disease behavior^†^	0.463	0.87 (0.59–1.27)				
Non-penetrating, non-stricturing (B1)		Reference		Reference		
Stricturing (B2)	0.079	2.18 (0.92–5.21)	0.671	1.41 (0.29–6.93)		
Penetrating (B3)	0.093	0.35 (0.10–1.20)	0.897	1.14 (0.16–8.37)		
Perianal disease at CD diagnosis	0.059	0.40 (0.16–1.03)	0.115	0.35 (0.09–1.29)		
History of GI surgery	0.043	0.35 (0.13–0.97)	0.240	0.43 (0.10–1.77)		
History of biological therapy	0.914	1.05 (0.42–2.61)				
Duration of remission induction with combination therapy	0.840	1.00 (0.98–1.03)				
Duration of remission on AZA monotherapy	0.762	1.08 (0.65–1.79)				
CDAI > 220	0.286	1.61 (0.67–3.8)				
BMI > 18 kg/m^2^	0.668	0.83 (0.35–1.96)				
ESR > 20 mm/h	0.117	5.06 (0.67–38.46)	0.512	2.07 (0.24–18.20)		
**CRP** **>** **10 mg/L**	**0.127**	**2.68 (0.76–9.46)**	**0.037**	**6.27 (1.11–35.13)**	**0.027**	**4.72 (1.19–18.75)**
WBC > 6.75 × 10^9^/L	0.154	1.92 (0.78–4.75)	0.368	1.81 (0.50–6.56)		
PLT > 300 × 10^9^/L	0.752	1.15 (0.48–2.74)				
Hemoglobin < 110 g/L	0.752	1.16 (0.47–2.86)				
AZA dosage ≥ 100 mg/d	0.795	1.00 (0.99–1.02)				
Thalidomide dosage ≥ 50 mg/d	0.371	1.01 (0.98–1.05)				
**6-TGN** **≥** **235 pmol/8** **× 10**^**8**^ **erythrocyte** **on AZA monotherapy**	**0.033**	**2.87 (1.04–7.92)**	**0.030**	**5.97 (1.19–30.03)**	**0.014**	**5.32 (1.40–20.14)**
6-TGN ≥ 235 pmol/8 × 10^8^ erythrocyte on combination therapy	0.062	0.36 (0.13–1.05)	0.411	0.63 (0.21–1.91)		

† *Phenotypes were categorized using the Montreal classification*.

*CD, Crohn's disease; BMI, body mass index; CDAI, Crohn's disease activity index; CRP, C-reactive protein; WBC, white blood cell; PLT, blood platelet; GI, gastrointestinal; AZA, azathioprine; TGN, thioguanine nucleotides; HR, hazard ratio; CI, confidence interval*.

#### Crohn Disease–Related Hospitalizations and Intestinal Surgery

A total of 9 (9.2%) patients were hospitalized. The probabilities of CD-related hospitalization were 2.5 and 9.6% at 6 and 12 months, respectively (Figure 3B). Of all the baseline factors, no risk factors were found to be associated with hospitalization either at univariate or multivariate analyses. Five (5.1%) patients on combination therapy underwent intestinal surgery. The cumulative percentage of intestinal surgery was 7.1% at 3 years (Figure 3C). No risk factors were found to be associated with intestinal surgery.

### Endoscopic Evaluation

**Figure 5:**
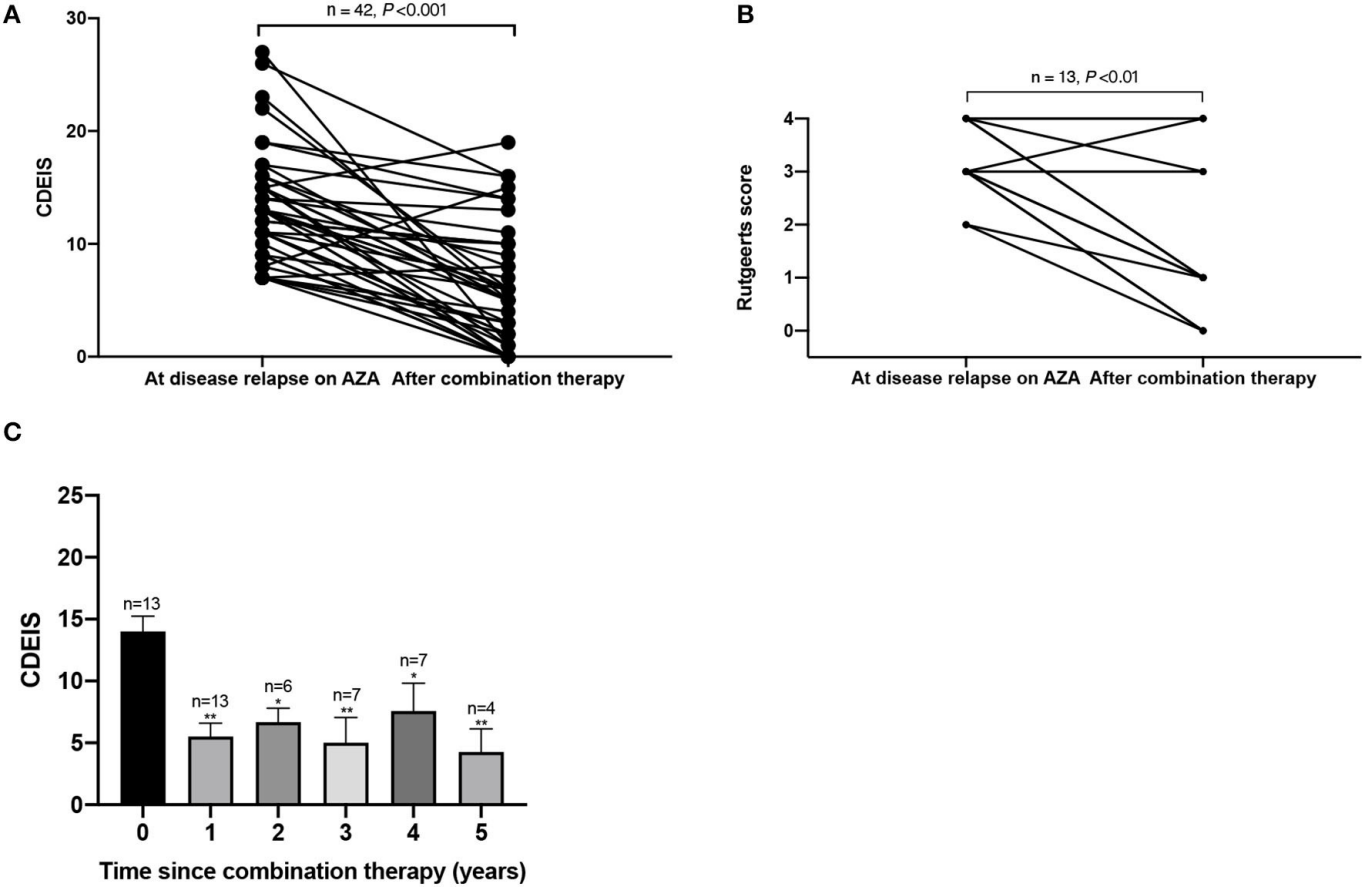
This figure shows the **(A)** CDEIS score and **(B)** Rutgeerts score before and after AZA and thalidomide combination therapy and the **(C)** trend of CDEIS in 13 patients during the 5-year follow-up. *P < 0.05 (compared with baseline), **P < 0.01 (compared with baseline). CDEIS, Crohn's Disease Endoscopic Index of Severity; AZA, azathioprine.

Fifty-five patients with endoscopic evaluation at the initiation of combination therapy underwent at least one endoscopy during follow-up. During follow-up, 35 (63.6%) patients achieved endoscopic remission and 13 (23.6%) patients achieved MH. Figures 5A,B showed that both CDEIS (13.40 ± 4.92 *vs*. 6.12 ± 5.24, *P* < 0.001) and Rutgeerts scores (3.23 ± 0.73 *vs*. 1.77 ± 1.59, *P* = 0.003) were significantly decreased on combination therapy. Figure 5C aimed to demonstrate the trend of CDEIS in 13 patients with multiple endoscopic examinations during the 5-year follow-up [mean ± standard deviation (SD) CDEIS were 14.00 ± 4.49; 5.49 ± 3.62; 6.67 ± 2.80; 5.00 ± 5.45; 7.57 ± 5.94; and 4.25 ± 3.77 at year 0, 1, 2, 3, 4, and 5, respectively]. The endoscopic disease activity was significantly decreased in the first year and remained stable during the follow-up.

### Adverse Events

A total of 90 AEs were observed in 50.8% (62/122) of the patients during the combination therapy (Table 4). Acroanesthesia was the most frequent AE (17.2% of the patients), followed by constipation (10.7%). In patients who experienced AEs, five discontinued AZA and seven discontinued thalidomide. The symptoms in all 13 patients were relieved after treatment discontinuation. The median AE-free duration was 13.1 months (IQR, 6.0–43.0 months) (Figure 3D). The cumulative percentages of patients free of AEs at 12, 24, 36, 48, and 60 months were 53.1, 39.1, 31.3, 22.4, and 15.0%, respectively. History of biological therapy (HR, 0.41; 95% CI, 0.20–0.83; *P* = 0.014) and presence of *NUDT15* variant (HR, 5.58; 95% CI, 1.02–65.9; *P* = 0.048) were associated with the occurrence of AEs at univariate analysis, but no independent risk factors were found in the multivariate analysis (Supplementary Table 1).

**Table 4:** Adverse events of thalidomide and AZA combination therapy.

	***n* = 122**
Patients with adverse events	62 (50.8%)
Events led to AZA discontinuation	5 (4.1%)
Events led to thalidomide discontinuation	7 (5.7%)
Events led to both AZA and thalidomide discontinuation	0
Events led to hospitalization	1 (0.8%)
Overall adverse events	90
Neuropathy	35 (28.7%)
Acroanesthesia	21 (17.2%)
Somnolence	8 (6.6%)
Vertigo	6 (4.9%)
Fatigue	2 (1.6%)
Insomnia	4 (3.3%)
Leukopenia	7 (5.7%)
Constipation	13 (10.7%)
Arthralgia	4 (3.3%)
Menstrual disorder	4 (3.3%)
Alopecia	2 (1.6%)
Pulmonary infection	2 (1.6%)
Blurred vision	5 (4.1%)
Tremors	3 (2.5%)
Chest distress	1 (0.8%)
Venous thromboembolism	1 (0.8%)
Dermatitis/erythra	2 (1.6%)
Varicella/shingles	3 (2.5%)
Lack of appetite	1 (0.8%)
Asymptomatic bradycardia	1 (0.8%)

*The values are described as number (%). AZA, azathioprine*.

## Discussion

To the best of our knowledge, our present study was the first to systematically evaluate the efficacy and safety of thalidomide and AZA combination therapy as a salvage therapy in AZA-refractory CD patients. Our study showed that thalidomide combined with AZA was effective in inducing clinical remission and sustaining long-term steroid-free remission in CD patients who lost response to AZA monotherapy.

When assessing loss of response to thiopurine monotherapy, an evaluation should be performed to identify and confirm the reasons. For patients who previously failed in thiopurine monotherapy due to skewed metabolism, low-dose thiopurine combined with allopurinol is a commonly applied optimization strategy to improve the efficacy of thiopurine (27). However, allopurinol therapy may not be suitable for patients who had a good initial response and tolerance to thiopurine but failed in the maintenance therapy. Since the use of thalidomide for the treatment of steroid-resistant CD in 1997 (28), the potential therapeutic role of thalidomide in refractory CD was investigated in several randomized controlled trials, open-label studies, and retrospective case series. A systematic review reported that thalidomide induced clinical remission in 51.5% of patients with CD (29). A prospective open-label cohort study assessed the efficacy of low-dose thalidomide (50–100 mg/d) in adult patients with active refractory CD. The clinical remission rates at weeks 8, 12, and 24 were 23.4, 46.8, and 53.2%, respectively (30). In the current study, the clinical remission rates at week 8, 12, and 24 were 30.3, 57.4, and 70.5%, respectively, which were relatively higher than in the previous study. Furthermore, these results may imply that the action of the combination therapy may take up to 8 weeks to be optimally appreciated. This time point is generally in agreement with or somewhat earlier than that of the previously reported optimal time frame for thalidomide monotherapy [8 weeks (15) and 12 weeks (30)].

Thalidomide is currently recommended only as a short-term therapy for refractory CD patients due to its relatively high incidence of AE. Therefore, only a few studies have investigated the long-term efficacy of thalidomide. A multicenter randomized clinical trial reported that the mean duration of clinical remission in children and adolescents with CD who had received thalidomide was 181.1 weeks (15). As for the adult CD, Simon et al. (13) reported that the probability of relapse was 37% at 6 months. A systematic review of 489 patients reported that thalidomide-maintained remission in 80.0 and 72.2% of the patients at 6 and 12 months, respectively (29). In the current study, the cumulative percentages of patients who maintained remission at 12, 24, 36, 48, and 60 months were 85.1, 78.3, 70.1, 66.6, and 55.8%, respectively. The median duration of remission was 74.7 months (95% CI, 39.6–109.9 months). Compared with previous studies on thalidomide, our study suggested that a longer duration and a higher clinical remission rate can be achieved with thalidomide and AZA combination therapy.

TNF-α is considered to be centrally involved in the inflammatory process in CD (31), and it is known that thiopurines have synergistic effects with anti-TNF-α drugs by reducing the immunogenicity of biological therapy and increasing the anti-TNF trough levels (32, 33). Thalidomide as an oral anti-inflammatory drug blocks TNF-α expression by various possible mechanisms (34, 35). Thus, we speculated that in addition to their own specific mechanism, combined thiopurines with thalidomide may have similar synergistic effects with anti-TNFα drugs. This was confirmed by our preliminary result showing that patients on combination therapy achieved long-term clinical remission. Given that both agents can modulate the function of T-cells (32, 34), the synergistic effects may also be *via* the modulation of T-cell immunity. However, the exact mechanism warrants further study.

A higher rate of AZA-related AEs, such as myelotoxicity, was observed in Asian patients with CD than in Caucasian patients (36, 37). Of the 98 patients on the AZA and thalidomide combination maintenance therapy, 35 patients had prior subtherapeutic TGN level, which paralleled the lower dose of AZA. These patients all had a good initial response to AZA monotherapy, but 25 patients experienced leukopenia and 10 patients experienced arthralgia. For safety concerns, we did not increase the AZA dosages for these 35 patients. Interestingly, our study showed that the risk of clinical relapse on combination therapy was higher for the other 63 patients who lost response despite having achieved the recommended therapeutic TGN concentrations. TGNs, which are active metabolites of AZA and 6-MP, function as rogue nucleic acids, disrupting the DNA replication in the most rapidly dividing cells such as activated T lymphocytes where the genes involved in T-cell immunity are downregulated by AZA (38). Thalidomide can reduce TNF-α and interleukin-12 production in patients with chronic active CD (39). Thus, activated T cell lymphocytes are targets for both AZA and thalidomide. If patients' diseases flared while their TGNs were within the therapeutic window, it means that some signaling pathways other than those of the T cell immunity may be playing an important role in triggering disease flare. Thus, the combined therapy with thalidomide may fail as well. This finding offered new insights into the solution of “maintenance therapy” in refractory CD patients with relatively poor tolerance to thiopurines. Considering that thiopurine failure due to intolerance occurs in up to 30% of recipients (40), in this setting, combination with thalidomide may present a promising alternative treatment. The TGN level with combination therapy was not found to be associated with disease relapse.

The present study also found elevated serum CRP levels at the initiation of the combination therapy as a risk factor for disease relapse. CRP level is related to a changed state of intestinal inflammation and is often used along with CDAI to assess IBD disease activity (41, 42). A high CRP level might be related to a high inflammatory burden at disease relapse (43). A retrospective study from Belgium concerning the long-term outcome of immunomodulator use indicates that a high disease burden at diagnosis predicted the need for step-up therapy using either biologics or surgery (44). CRP can also quickly reflect the effectiveness of drugs, thereby guiding the clinical treatment (45). In our study, there was a significant decrease in CRP (*P* = 0.032) along with CDAI (*P* < 0.001) at week 8 compared to the baseline.

The treatment goal of CD has evolved from symptom control toward MH (46). A retrospective study reported that a positive endoscopic response and MH were documented in 46 and 38% of patients treated with thiopurines at 12 months, respectively (47). As for studies on thalidomide, He et al. (30) reported that the endoscopic remission and MH rates were 43.8 and 28.1% in patients with CD, respectively. A long-term study reported that 52 weeks of treatment with thalidomide led to MH in 20 (27.7%) of 70 patients with IBD (48). The current study showed that the endoscopic remission and MH rates in patients treated with the combination of AZA and thalidomide were 63.6 and 23.6%, respectively. Compared with previous studies, the present study had a relatively lower MH rate. This may be partly due to the large percentage of enrolled patients with complicated cases (46.7% were with stricturing disease and 23.8% had penetrating lesion at diagnosis) that were refractory to AZA.

Although AEs are associated with both AZA and thalidomide, the incidence of AEs reported in the current study was similar to that of previous studies (5, 14, 29, 49); indicating that the combination of AZA and thalidomide may not increase the risk of AEs. Teratogenicity is the most well-known AE of thalidomide (50), but it is also the most preventable one. A retrospective study reviewed 124,000 (43% female) patients receiving thalidomide in 6 years to determine the occurrence of positive pregnancy tests whilst on treatment. Only one female was pregnant while on thalidomide and the pregnancy resulted in a miscarriage (51). No data from human studies are available on how breastfeeding during treatment with thalidomide might affect the infant. However, owing to the potential toxicity, thalidomide is absolutely contraindicated during lactation (52). In the current study, all patients were fully informed of the risk of teratogenicity, with the mandatory requirement for contraception throughout the duration of the therapy and up to 6 months after thalidomide had been discontinued.

There are a few limitations to our study. First, our findings should be interpreted with caution due to the retrospective and referral center-based design of our investigation. However, most of the data were collected prospectively and had been structurally documented in the patients' medical files during each follow-up, which minimized these biases. We believe that our results reflect the clinical effectiveness of AZA and thalidomide combination therapy in real-world clinical practice. Furthermore, one may argue that biologic agents could be used in the event of loss of response to AZA. So far, only two biologic agents (infliximab and adalimumab) are approved for CD in China and both are not covered by the health insurance till January 1, 2020. In the current study, one-third (33, 27.0%) of included patients had previously failed on one of these two biologics and the remaining two-third patients could not afford to use biologics in the long-term. The current study aimed to offer an alternative option for patients who lost response to AZA and were unable to tolerate or could not afford biologics.

In conclusion, thalidomide combined with AZA is effective in inducing and maintaining clinical remission and is well-tolerated in CD patients who lost response to AZA monotherapy. Moreover, MH and endoscopic remission were achieved 23.6 and 63.6% of patients. Further well-designed prospective study with control is needed to validate the conclusions drawn from our study.

## Data Availability Statement

The raw data supporting the conclusions of this article will be made available by the authors, without undue reservation.

## Ethics Statement

The studies involving human participants were reviewed and approved by The Ethics Committee of the First Affiliated Hospital of Sun Yat-sen University. Written informed consent to participate in this study was provided by the participants' legal guardian/next of kin.

## Author Contributions

TL, YQ, and XL were responsible for the study concept and design, acquisition of data, analysis, and interpretation of data. TL and YQ wrote the manuscript. ML, XZ, SH, RF, and BC contributed to the collection of data. YH and ZZ contributed to the revision of the manuscript. MC and SZ contributed to the design and conceptualization of the research, coordination of the research as lead investigator, and revision of the manuscript. All authors approved the final version of the submitted manuscript and have agreed to be accountable for all aspects of the work.

## Conflict of Interest

The authors declare that the research was conducted in the absence of any commercial or financial relationships that could be construed as a potential conflict of interest.
