# Supplementary material for: Thalidomide Combined With Azathioprine as Induction and Maintenance Therapy for Azathioprine-Refractory Crohn's Disease Patients
Source: Front Med (Lausanne). 2020 Nov 6;7:557986. doi: 10.3389/fmed.2020.557986 (PMC7677527; doi:10.3389/fmed.2020.557986)
Supplement: Supplementary file 1 [file Table_1.DOCX]

Supplementary Material

**Supplementary Table 1.** Predictors of adverse events by univariable analysis (log-rank test) and multivariable analysis (Cox model)

| Factors | Univariate |  |  | Multivariate |  |
| --- | --- | --- | --- | --- | --- |
|  | *P* | HR (95% CI) |  | *P* | HR (95% CI) |
| Gender, female | 0.076 | 1.58 (0.95-2.63) |  |  |  |
| Age, >40 years | 0.673 | 0.87 (0.33-1.70) |  |  |  |
| Smoker | 0.955 | 1.0 (0.99-1.01) |  |  |  |
| Disease duration |  |  |  |  |  |
| Disease location † |  | 1.00 |  |  |  |
| Ileal (L1) | 0.626 | 1.29 (0.47-3.56) |  |  |  |
| Colonic (L2) | 0.014 | 0.41 (0.20-0.83) |  |  |  |
| Ileocolonic (L3) | 0.547 | 0.78 (0.35-1.72) |  |  |  |
| Upper digestive tract (L4) |  |  |  |  |  |
| Disease behavior † |  | 1.00 |  |  |  |
| Nonpenetrating, nonstricturing (B1) | 0..413 | 1.26 (0.72-2.22) |  |  |  |
| Stricturing (B2) | 0.756 | 1.12 (0.54-2.34) |  |  |  |
| Penetrating (B3) | 0.008 | 0.46 (0.26-0.82) |  |  |  |
| Perianal disease at CD diagnosis | 0.076 | 1.58 (0.95-2.63) |  |  |  |
| History of intestinal surgery | 0.125 | 0.68 (0.42-1.11) |  |  |  |
| History of biological therapy | 0.014 | 0.41 (0.20-0.83) |  |  |  |
| Duration of remission on AZA monotherapy | 0.955 | 1.00 (1.00-1.01) |  |  |  |
| CDAI >220 | 0.974 | 0.99 (0.63-1.57) |  |  |  |
| BMI >18 kg/m^2^ | 0.686 | 0.91 (0.58-1.44) |  |  |  |
| ESR >20mm/h | 0.113 | 0.64 (0.37-1.11) |  |  |  |
| CRP >10mg/L | 0.768 | 1.11 (0.55-2.27) |  |  |  |
| WBC >6.75×10^9^/L | 0.195 | 0.72 (0.44-1.18) |  |  |  |
| PLT >318×10^9^/L | 0.875 | 1.04 (0.65-1.65) |  |  |  |
| HB <110g/L | 0.882 | 1.04 (0.62-1.74) |  |  |  |
| AZA dosage ≥100mg/d | 0.067 | 0.99 (0.98-1.00) |  |  |  |
| Thalidomide dosage ≥50mg/d | 0.361 | 1.01 (0.99-1.03) |  |  |  |
| 6-TGN <235 pmol/8 × 10^8^ erythrocyte on combination therapy | 0.869 | 1.05 (0.62-1.75) |  |  |  |
| NUDT15 variant | 0.048 | 5.58 (1.02-65.9) |  |  |  |

† Phenotypes were categorized using the Montreal classification.

CD, Crohn’s disease; BMI, body mass index; CDAI, Crohn’s disease activity index; CRP, C-reactive protein; HB, hemoglobin; WBC, white blood cell; PLT, blood platelet; AZA, azathioprine; TGN, thioguanine nucleotides; NUDT, nudix hydrolase; HR, hazard ratio; CI, confidence interval.
